# Supplementary material for: Normal breast tissue (NBT)-classifiers: advancing compartment classification in normal breast histology
Source: NPJ Breast Cancer. 2026 Feb 9;12:41. doi: 10.1038/s41523-026-00896-2 (PMC12996451; doi:10.1038/s41523-026-00896-2)
Supplement: Supplementary file 1 — NPJ_BreastCancer_2fd7f8ba-044e-423c-89b3-ff07ca907853_Supplementary_Material_Revision_clean [file 41523_2026_896_MOESM1_ESM.pdf]

Table of Contents

Supplementary Figures 1

Supplementary Fig. 1 Schematic pipeline of the manual annotation procedure using QuPath v0.3.0. 1

Supplementary Fig. 2 Illustration of the histology of NBTs captured at different fields of view. 2

Supplementary Fig. 3 Examples of annotated WSIs of NBTs from BCI, EPFL, NKI, SGK and KHP cohorts. 3

Supplementary Fig. 4 Illustration of *NBT-Classifiers'* architecture. 4

Supplementary Fig. 5 Illustration of different stain normalisation methods. 5

Supplementary Fig. 6 Barplot showing the number of patches obtained using different patch sizes. 6

Supplementary Fig. 7 Sample workflow for three-fold cross-validation experiments. 7

Supplementary Fig. 8 Confusion matrices evaluating the 224px-, 512px and 1024px-based *NBT-Classifiers* on external datasets 8

Supplementary Fig. 9 Cohort-specific multi-class receiver operating characteristic curves for the 512px-based *NBT-Classifier*. 8

Supplementary Fig. 10 Cohort-specific multi-class ROC curves for the 1024px-based *NBT-Classifier*. 9

Supplementary Fig. 11 Boxplot comparisons of *NBT-Classifiers'* performance across different age groups and NBT sources. 9

Supplementary Fig. 12 t-SNE visualisation of features extracted by the 1024px-based *NBT-Classifier*. 10

Supplementary Fig. 13 Illustration of class activation mapping and gradient-weighted class activation mapping visualisations. 11

Supplementary Fig. 14 Additional CAM and Grad-CAM visualisations. 12

Supplementary Fig. 15 Consistent histopathological patterns are captured across multiple cohorts. 13

Supplementary Fig. 16 t-SNE visualisation of features extracted by the 512px-based *NBT-Classifier*. 14

Supplementary Fig. 17 CAM-based interpretation of low-confidence predictions by the 512px-based *NBT-Classifier*. 14

Supplementary Fig. 18 Proportional confusion matrices for the 1024px-based *NBT-Classifier* and HistoROI. 15

Supplementary Fig. 19 Representative regions with overlaid tissue probability heatmaps 16

Supplementary Fig. 20 Illustration of foreground tissue detection using HistoQC. 17

Supplementary Fig. 21 Examples illustrating the proposed *NBT-Classifier*-based WSI pre-processing pipeline. 18

Supplementary Fig. 22 t-SNE visualisation of feature embeddings illustrating domain shift across cohorts. 18

Supplementary Tables 19

Supplementary Table 1. Overview of annotated WSI datasets of NBTs 19

Supplementary Table 2. Summary of annotated patch-level datasets. 21

Supplementary Table 3. Summary of three-fold cross validation accuracies. 22

Supplementary Table 4. A summary of studies of NBTs with manual annotations in recent ten years 23

Supplementary Figures

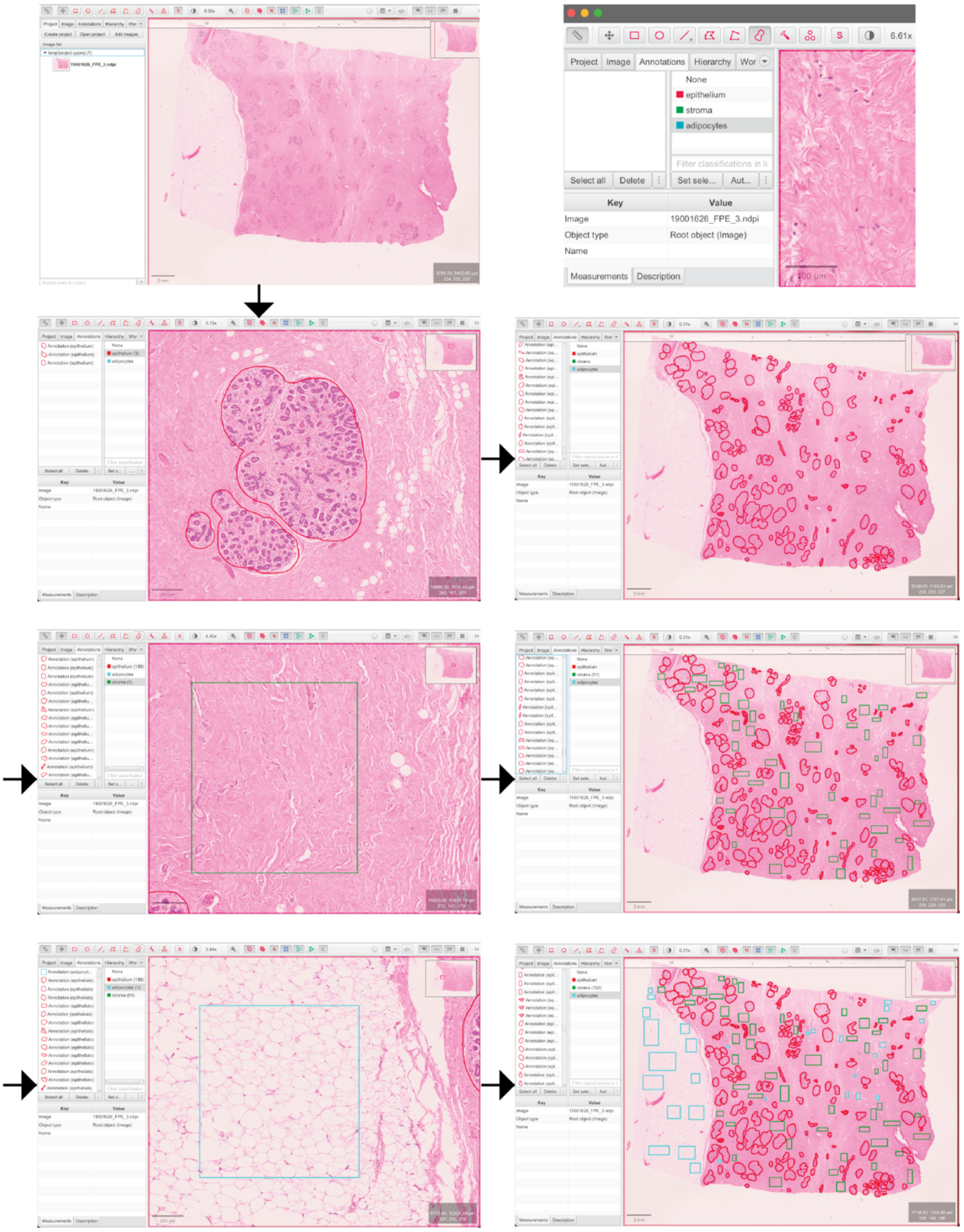

Supplementary Fig. 1 Schematic pipeline of the manual annotation procedure using QuPath v0.3.0. For each WSI, epithelium regions (in red) were exhaustively delineated using the brush tool to ensure precise annotation. To obtain a balanced number of training patches from each tissue class, similarly sized regions of stroma (in green) and adipocytes (in blue) were randomly selected from various areas of the WSI using rectangular boxes. This approach enhances efficiency while ensuring the inclusion of spatial variations and maintaining proportional representation across tissue types.

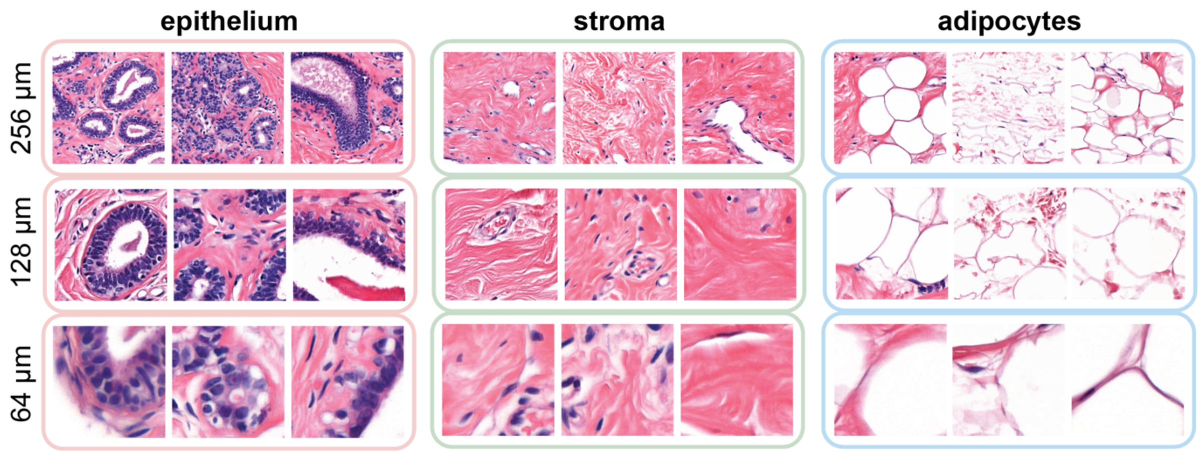

Supplementary Fig. 2 Illustration of the histology of NBTs captured at different fields of view. From left to right, the columns display example patches of epithelium, stroma, and adipocytes. Within each column, the rows, from top to bottom, show histological structures captured at fields of view of 256 x 256 μm, 128 x 128 μm, and 64 x 64 μm, respectively.

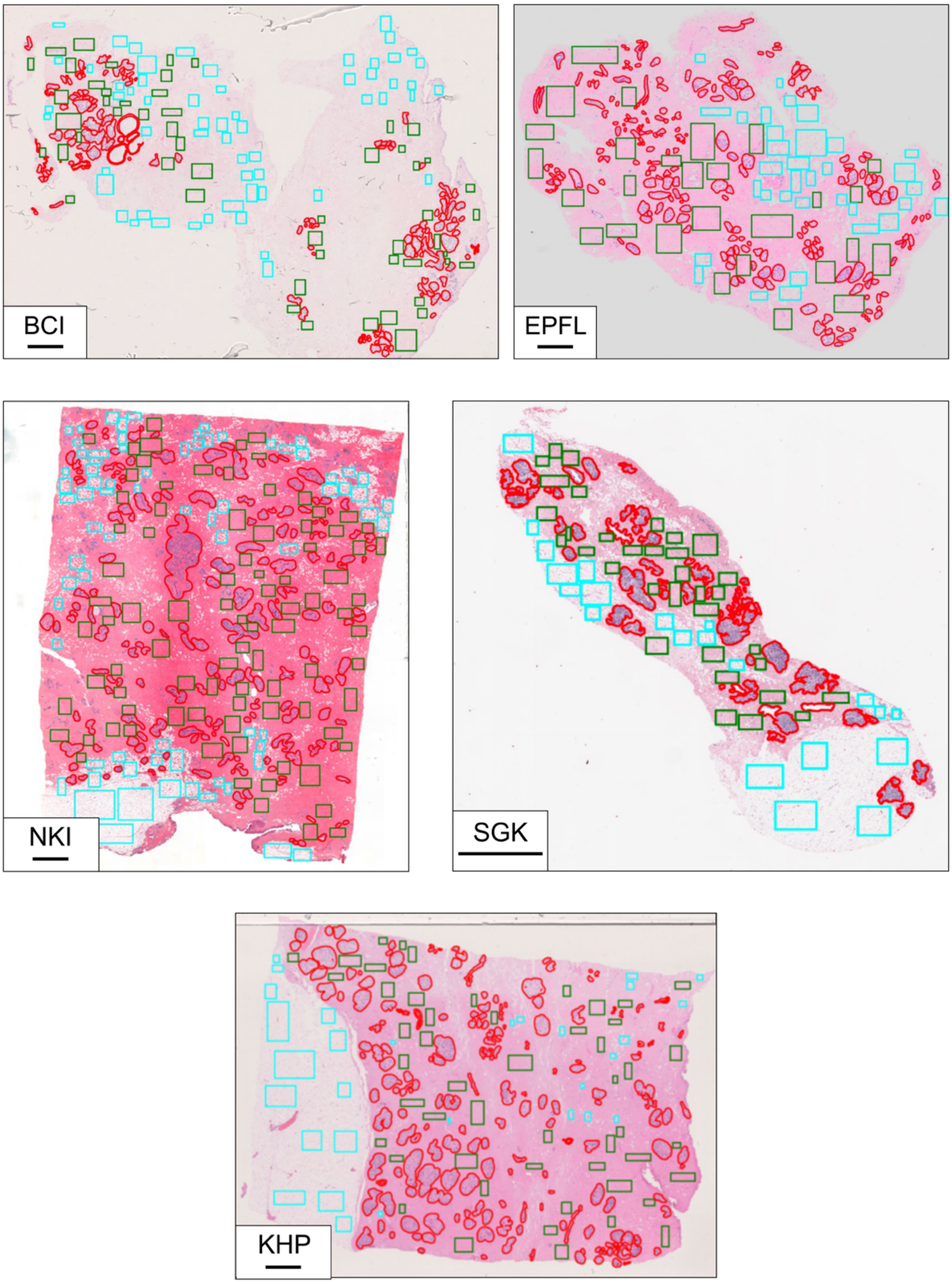

Supplementary Fig. 3 Examples of annotated WSIs of NBTs from BCI, EPFL, NKI, SGK and KHP cohorts. Epithelium, stroma, and adipocytes are annotated in red, green, and blue, respectively. Scale bar, 2 mm.

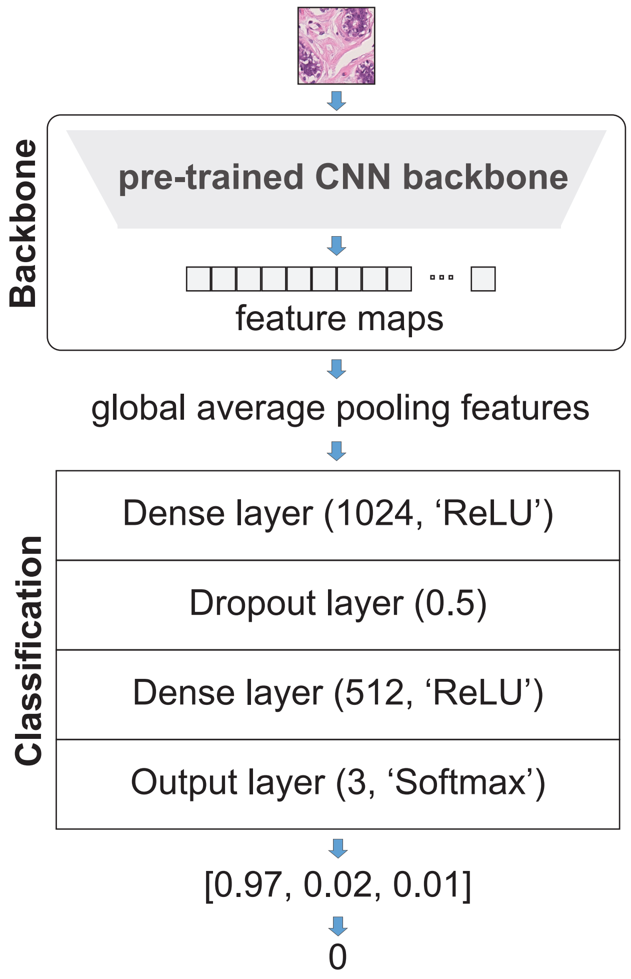

Supplementary Fig. 4 Illustration of *NBT-Classifiers'* architecture. Each *NBT-Classifier* utilises a pre-trained CNN backbone to extract feature maps, which are then processed via global average pooling. A classification head subsequently predicts the probabilities for epithelium, stroma, and adipocytes, with the final tissue class assigned based on the highest probability: 0 for epithelium, 1 for stroma, and 2 for adipocytes.

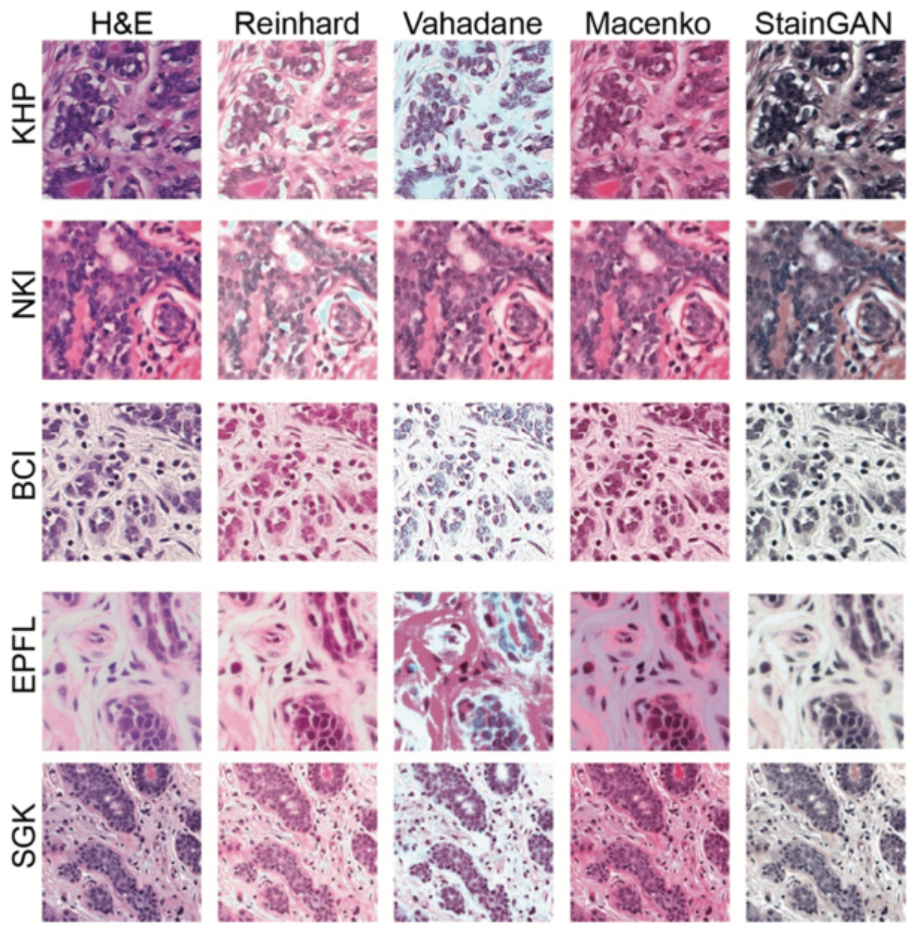

Supplementary Fig. 5 Illustration of different stain normalisation methods. The first column shows the original H&E patch images, highlighting considerable staining variations across KHP, NKI, BCI, EPFL and SGK cohorts. Columns two through five display the patches after normalisation using the Reinhard, Vahadane, Macenko, and StainGAN methods, demonstrating varying abilities to mitigate staining variations.

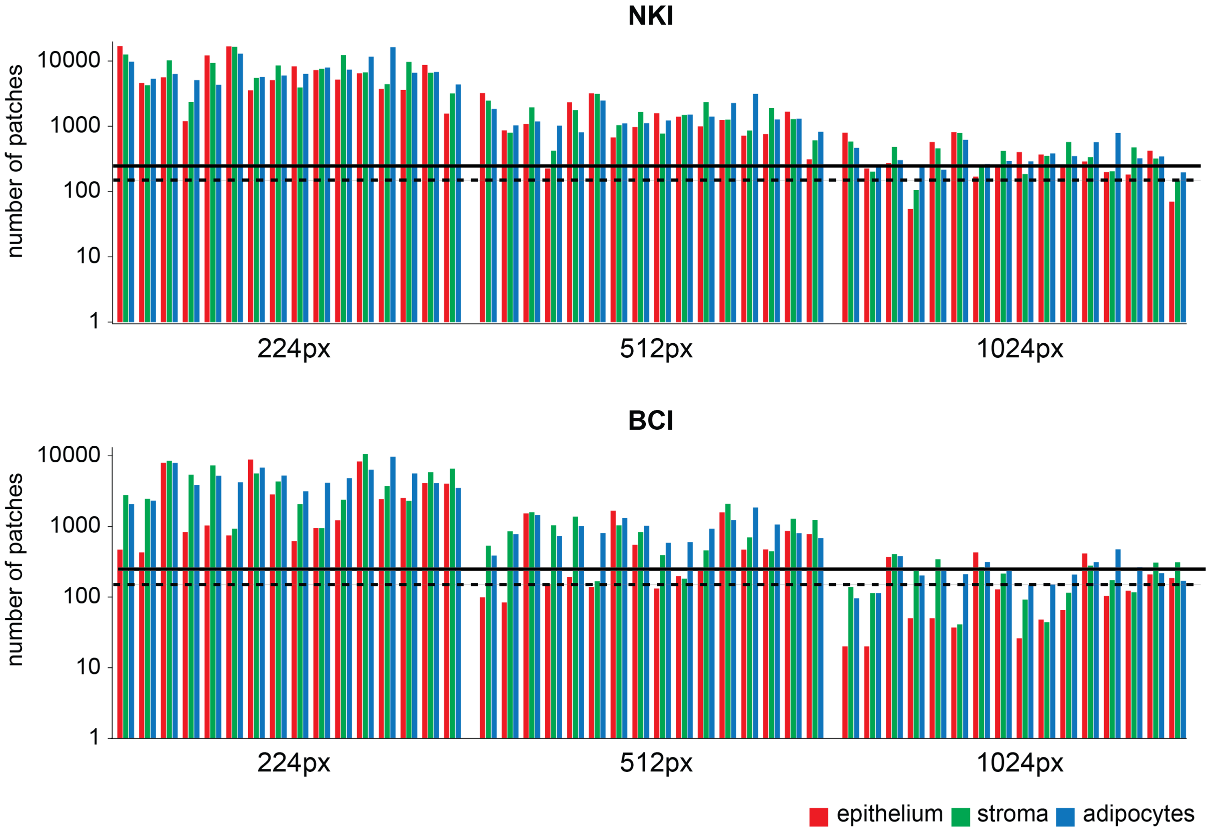

Supplementary Fig. 6 Barplot showing the number of patches obtained using different patch sizes. The panels illustrate the variation in the number of patches obtained from the NKI (top) and BCI (bottom) cohorts, using patch sizes of 224 x 224 pixels (denoted as 224px) (left), 512 x 512 pixels (denoted as 512px) (middle), and 1024 x 1024 pixels (denoted as 1024px) (right) at 40x magnification. In each panel, the x-axis represents individual WSI, with the coloured bar plots separately showing the number of epithelium (red), stroma (green), and adipocytes (blue) patches generated from each WSI at the corresponding patch size. The horizontal line indicates the number of 250 patches, while the horizontal dashed line indicates the number of 150 patches.

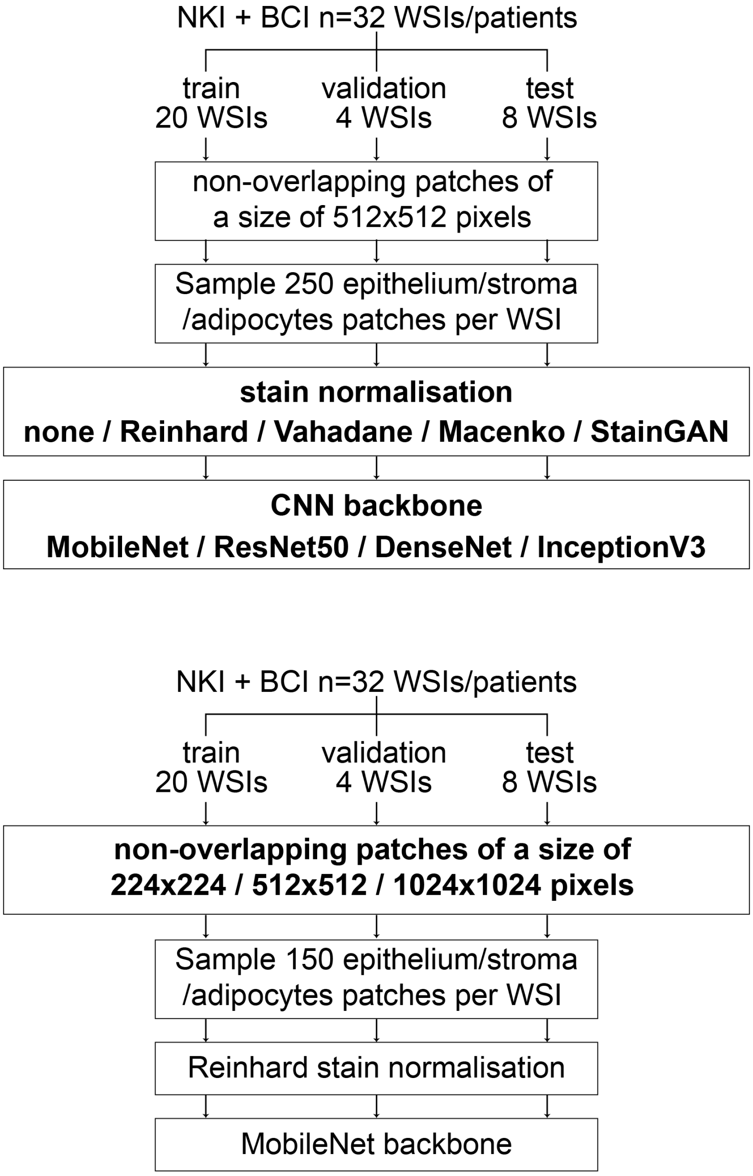

Supplementary Fig. 7 Sample workflow for three-fold cross-validation experiments. A total of 23 training configurations were evaluated, varying in stain normalisation methods, CNN backbones, and patch sizes.

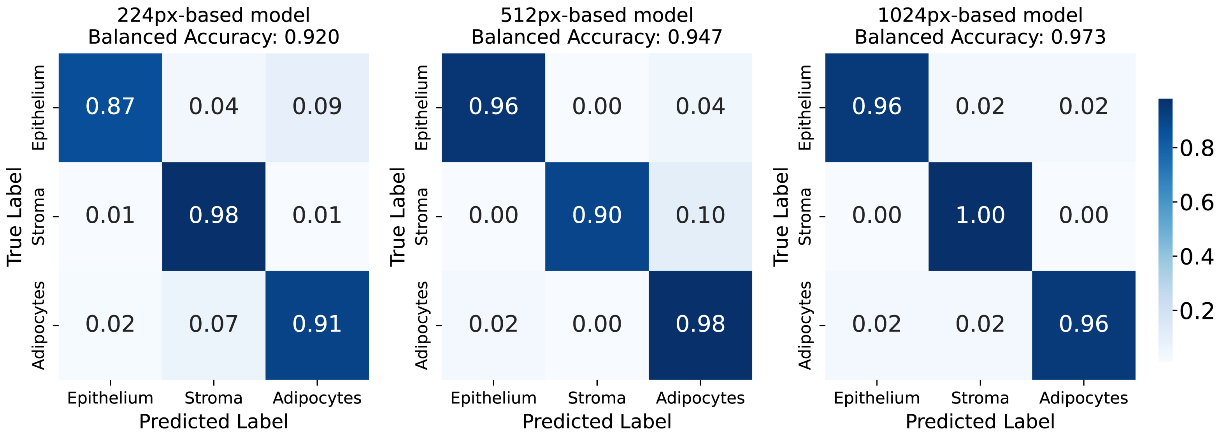

Supplementary Fig. 8 Confusion matrices evaluating the 224px-, 512px and 1024px-based *NBT-Classifiers* on external datasets. Each confusion matrix summarizes the classification results across the three external cohorts (KHP, EPFL, and SGK), comparing the predicted tissue classes: epithelium, stroma, and adipocytes (columns) against the ground-truth labels (rows). Diagonal cells indicate the number of correct predictions for each class, while off-diagonal cells indicate misclassifications. The colour intensity of each cell reflects the classification frequency, with lighter shades indicating higher counts. The overall balanced accuracy is derived from these matrixes and displayed at the top.

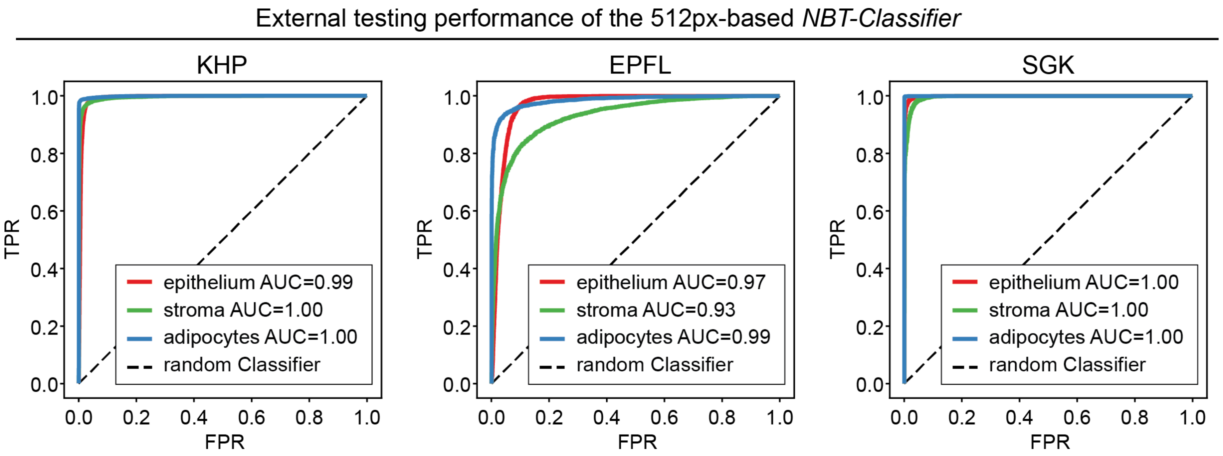

Supplementary Fig. 9 Cohort-specific multi-class receiver operating characteristic curves for the 512px-based *NBT-Classifier*. Panels from left to right show the receiver operating characteristic (ROC) curves of the 512px-based *NBT-Classifier* evaluated in the KHP, EPFL and SGK cohort, respectively. Performance of epithelium, stroma and adipocytes are indicated in red, green, and blue, respectively, with corresponding values of area under the ROC curve (AUC) presented below each curve.

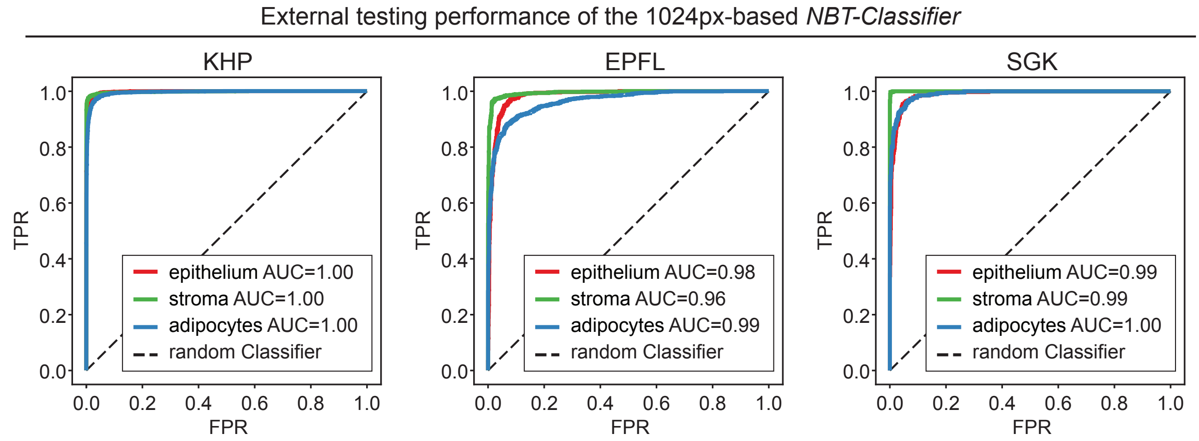

Supplementary Fig. 10 Cohort-specific multi-class ROC curves for the 1024px-based *NBT-Classifier*. Panels from left to right show the ROC curves of the 1024px-based *NBT-Classifier* evaluated in the KHP, EPFL and SGK cohort, respectively. Performance of epithelium, stroma and adipocytes are indicated in red, green, and blue, respectively, with corresponding values of AUC presented below each curve.

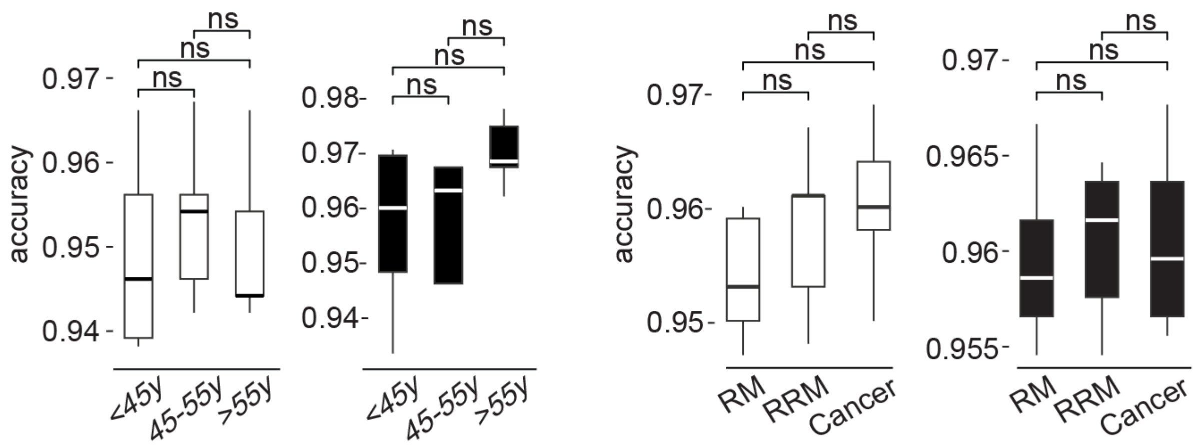

Supplementary Fig. 11 Boxplot comparisons of *NBT-Classifiers'* performance across different age groups and NBT sources. The performance of the 512px- and 1024px-based models is depicted in white and black boxplots, respectively. In each boxplot, the central line indicates the median, while the whiskers extend to the minimum and maximum values. The label "ns" denotes statistical non-significance, with adjusted p-values less than 0·05 considered significant. RM stands for reduction mammoplasty, and RRM stands for risk-reducing mastectomy.

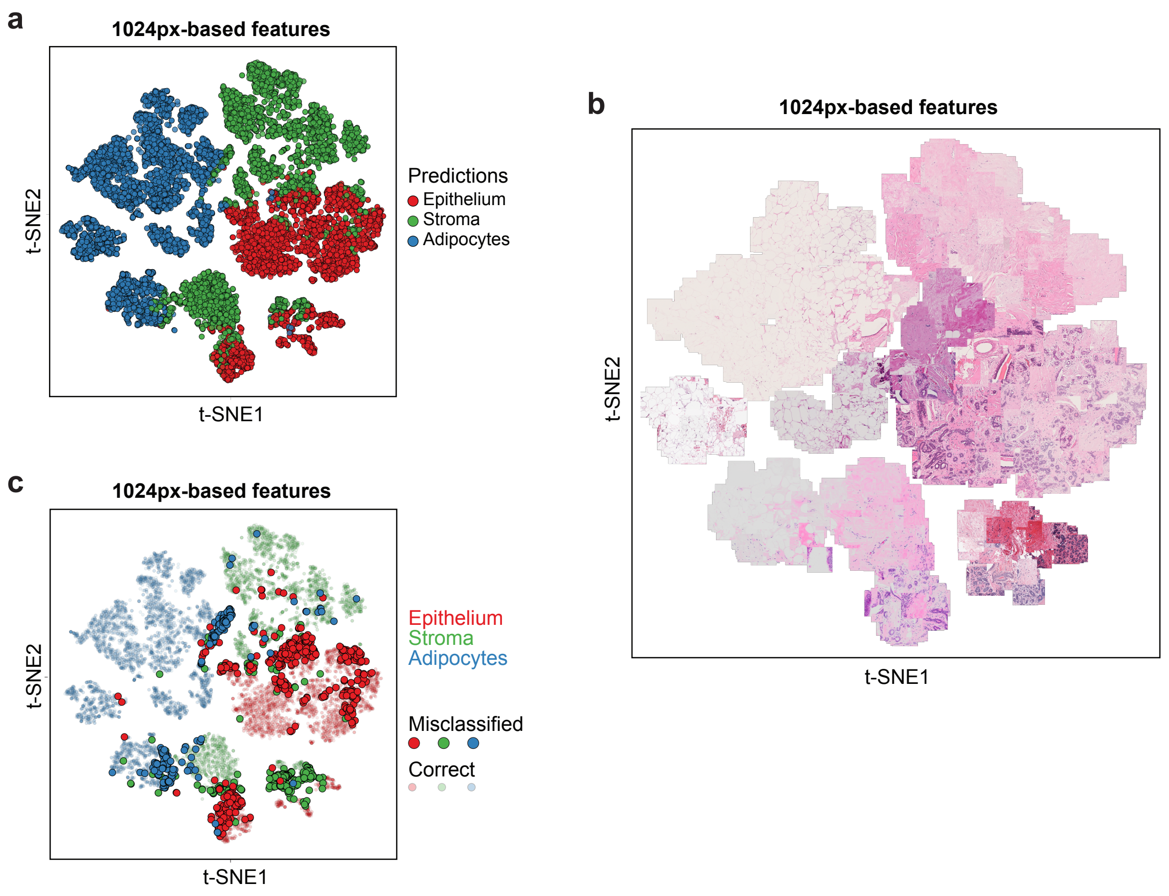

Supplementary Fig. 12 t-SNE visualisation of features extracted by the 1024px-based *NBT-Classifier*. Features are derived from the last layer (the layer before the output layer) of the 1024px-based *NBT-Classifier*. Panel **a** displays a t-Distributed Stochastic Neighbour Embedding (t-SNE) plot coloured by the 1024px-based *NBT-Classifier's* predictions. Panel **b** overlays original H&E-stained patches onto a subset of random features (500 per tissue class) on the t-SNE plot. Panel **c** displays a t-SNE plot with points coloured according to ground-truth tissue classes. Misclassified patches are marked as opaque circles (alpha=1.0), while correctly classified patches are represented by semi-transparent circles (alpha=0.3).

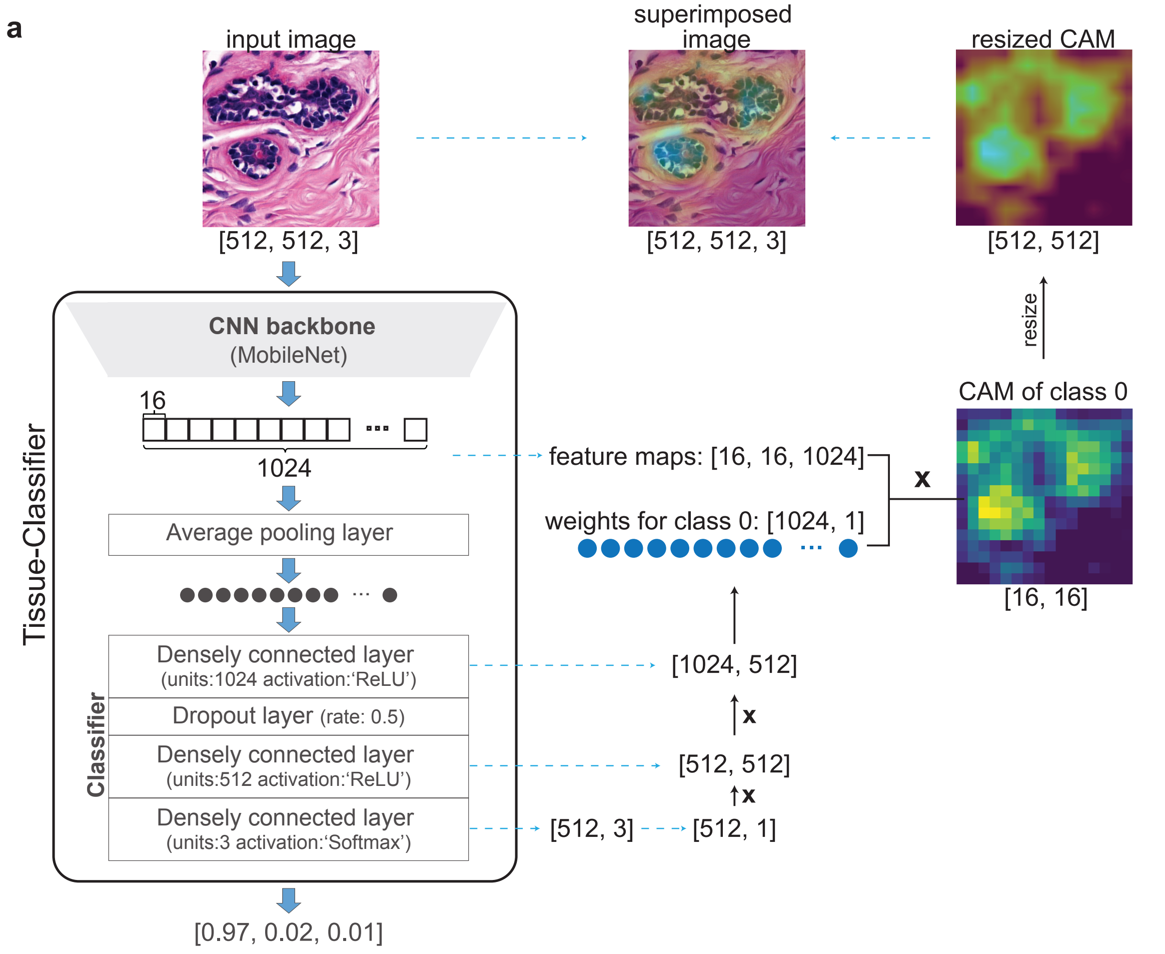

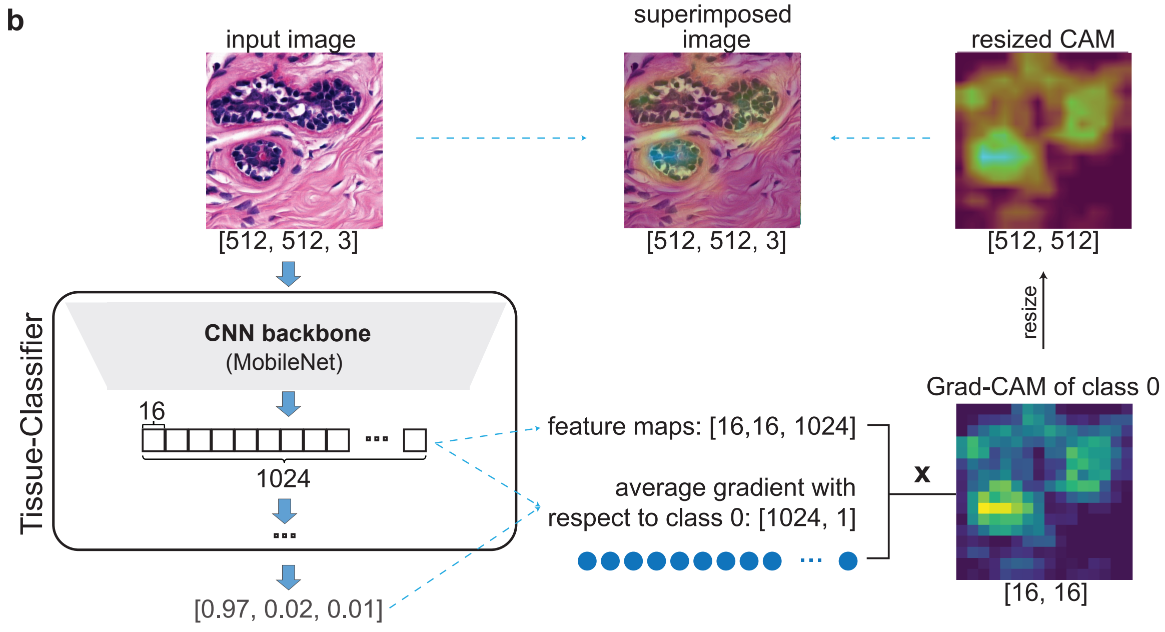

Supplementary Fig. 13 Illustration of class activation mapping and gradient-weighted class activation mapping visualisations. Class activation mapping (CAM) (**a**) uses class-specific weights to aggregate feature maps from the last convolutional layer of the CNN backbone, while gradient-weighted class activation mapping (Grad-CAM) (**b**) computes the average gradients. Both methods highlight high-attention regions that are relevant to a specific tissue class.

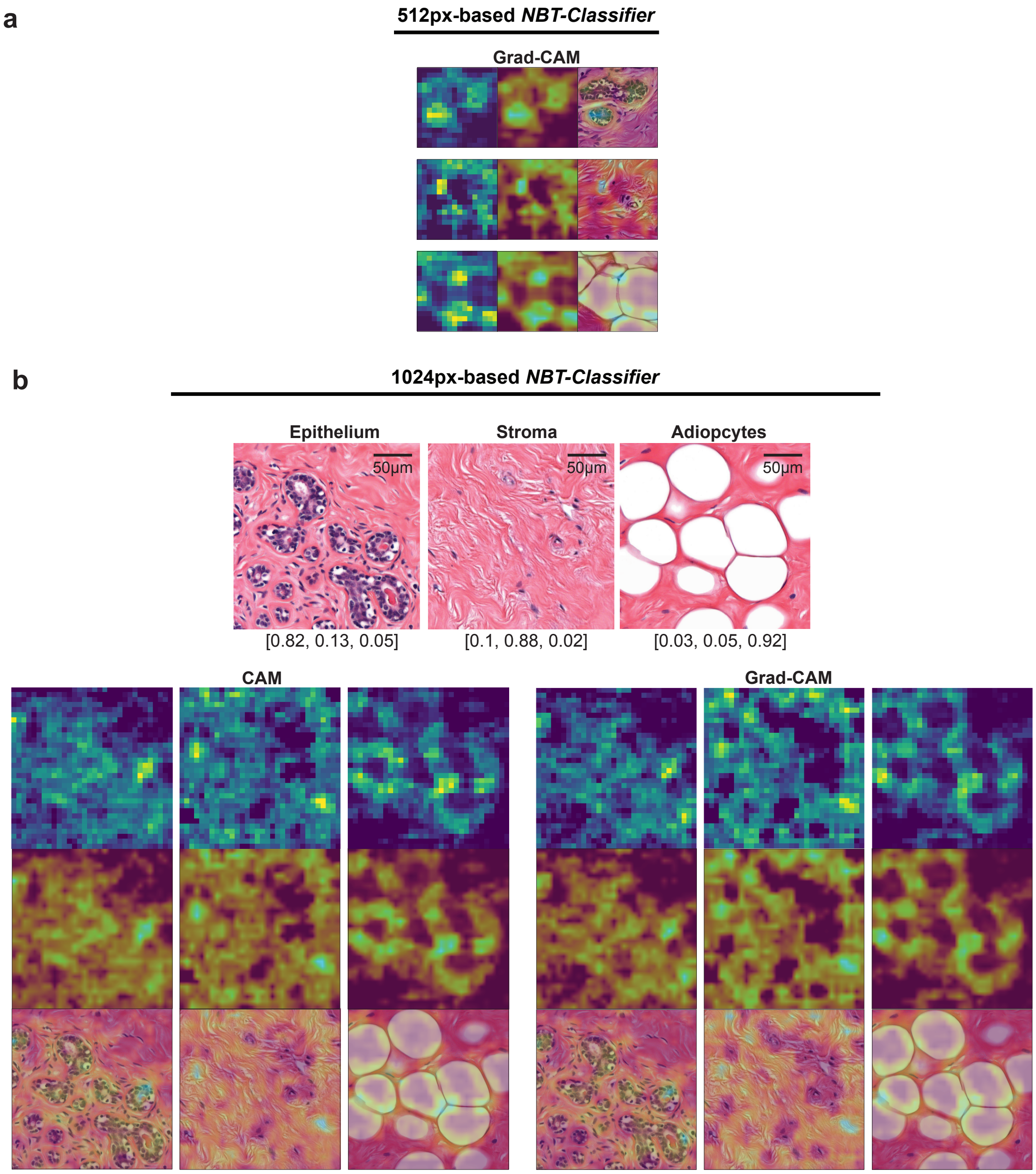

Supplementary Fig. 14 Additional CAM and Grad-CAM visualisations. Panel **a** displays Grad-CAM heatmaps for 512 x 512-pixel patches (0·25 µm/pixel) of epithelium (top row), stroma (middle row), and adipocytes (bottom row). Panel **b** shows original 1024 x 1024-pixel H&E patches (0·25 µm/pixel) and predictions (top), CAM (bottom left) and Grad-CAM (bottom right) visualisations. Each row, from left to right, includes the original CAM or Grad-CAM heatmaps, resized heatmaps, and heatmaps overlaid on the original patches.

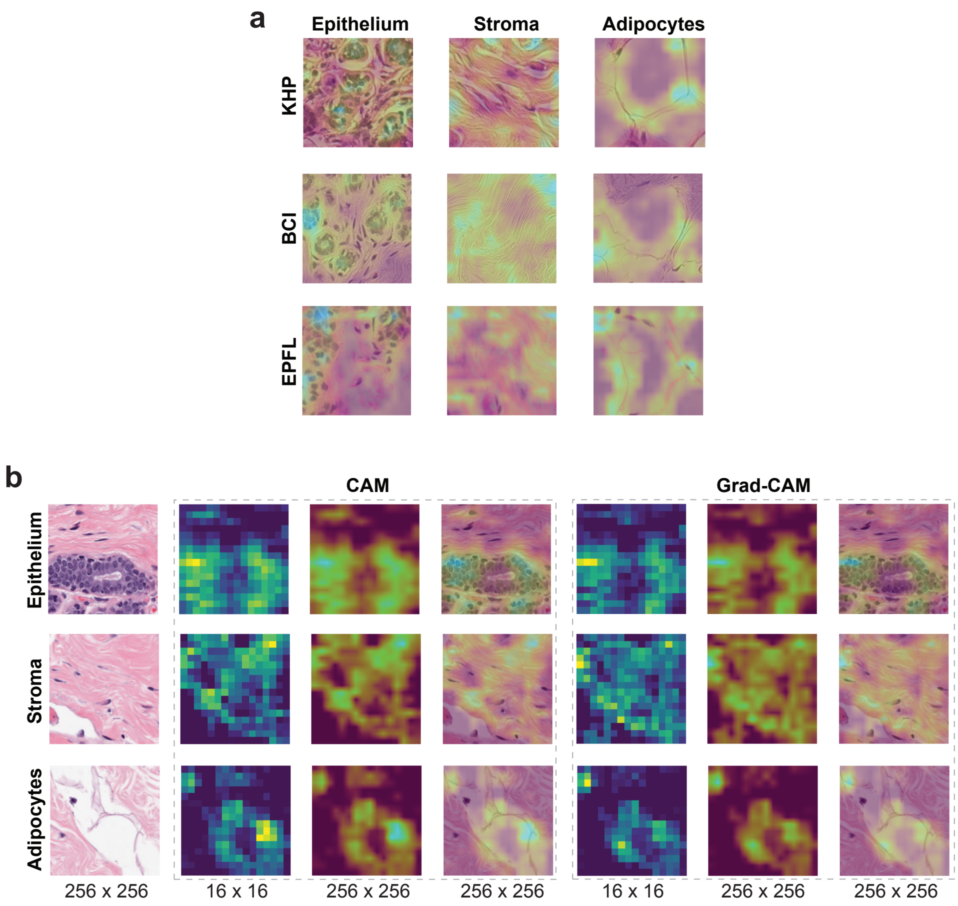

Supplementary Fig. 15 Consistent histopathological patterns are captured across multiple cohorts. In panel **a**, rows indicate CAM heatmaps overlaid on the Reinhard normalised epithelium, stroma and adipocytes patches (512 x 512 pixels, 0·25 µm/pixel) from KHP, BCI and EPFL cohorts. Each patch was predicted by the 512px-based *NBT-Classifier* with a probability of 1·0 of the predicted tissue class. The highlighted histopathological patterns in each tissue class are consistent across multiple cohorts. In panel **b**, patches are 256 x 256 pixels, 0·5 µm/pixel, obtained from WSIs in the SGK cohort that were scanned at 20x magnification, with CAM (left) and Grad-CAM (right) visualisations.

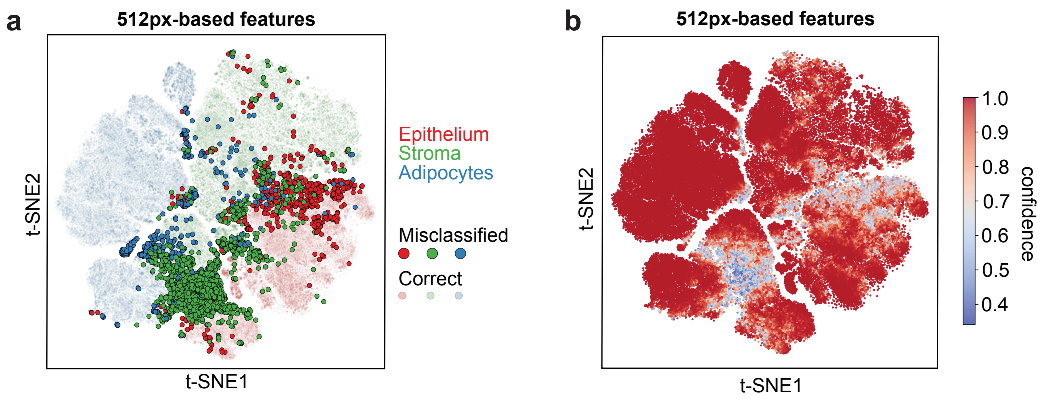

Supplementary Fig. 16 t-SNE visualisation of features extracted by the 512px-based *NBT-Classifier*. Features are derived from the last layer (just before the output layer) of the model. Panel **a** displays a t-SNE plot coloured by ground-truth tissue classes. Misclassified patches are marked as opaque circles (alpha=1.0), while correctly classified patches are represented by semi-transparent circles (alpha =0.3). Panel **b** presents the t-SNE visualisation of prediction confidence for the 512px-based *NBT-Classifier*. Each patch is coloured according to the probability of its predicted tissue class.

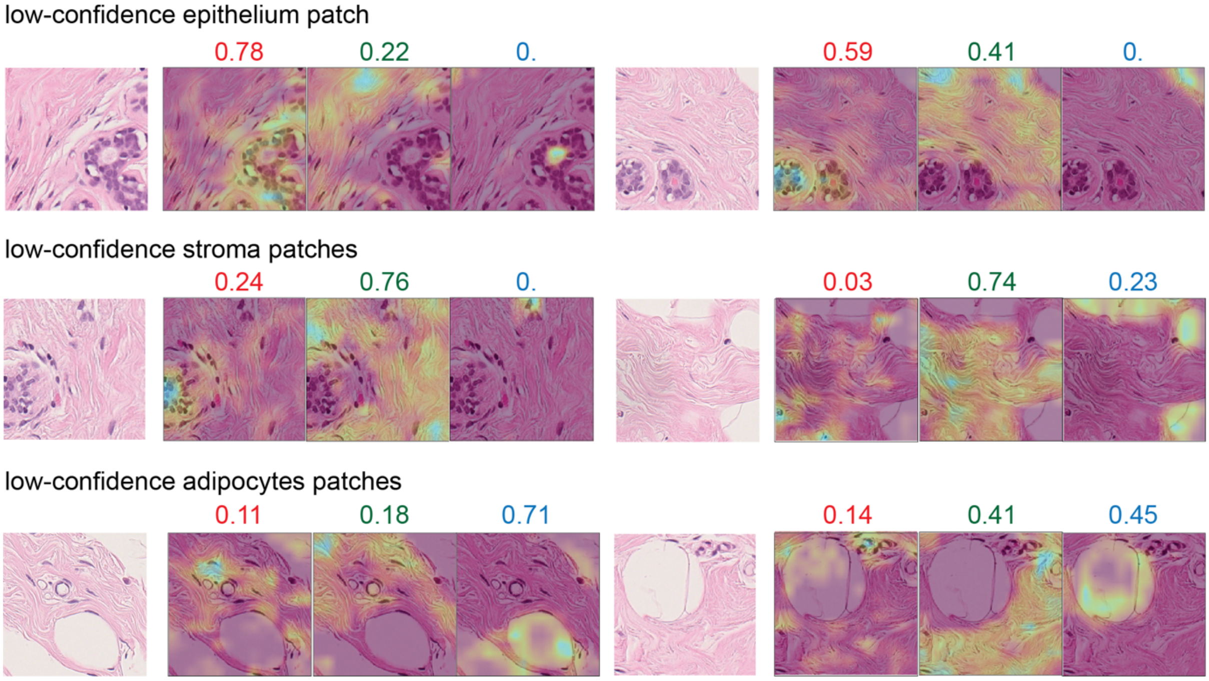

Supplementary Fig. 17 CAM-based interpretation of low-confidence predictions by the 512px-based *NBT-Classifier*. The rows, from top to bottom, show low-confidence 512 x 512-pixel patches predicted as epithelium, stroma, and adipocytes, respectively. For each patch, CAM heatmaps are generated for all three tissue classes and overlaid on the corresponding H&E patch image, highlighting class-specific high-attention regions. The predicted class probabilities are displayed at the top, with epithelium in red, stroma in green, and adipocytes in blue.

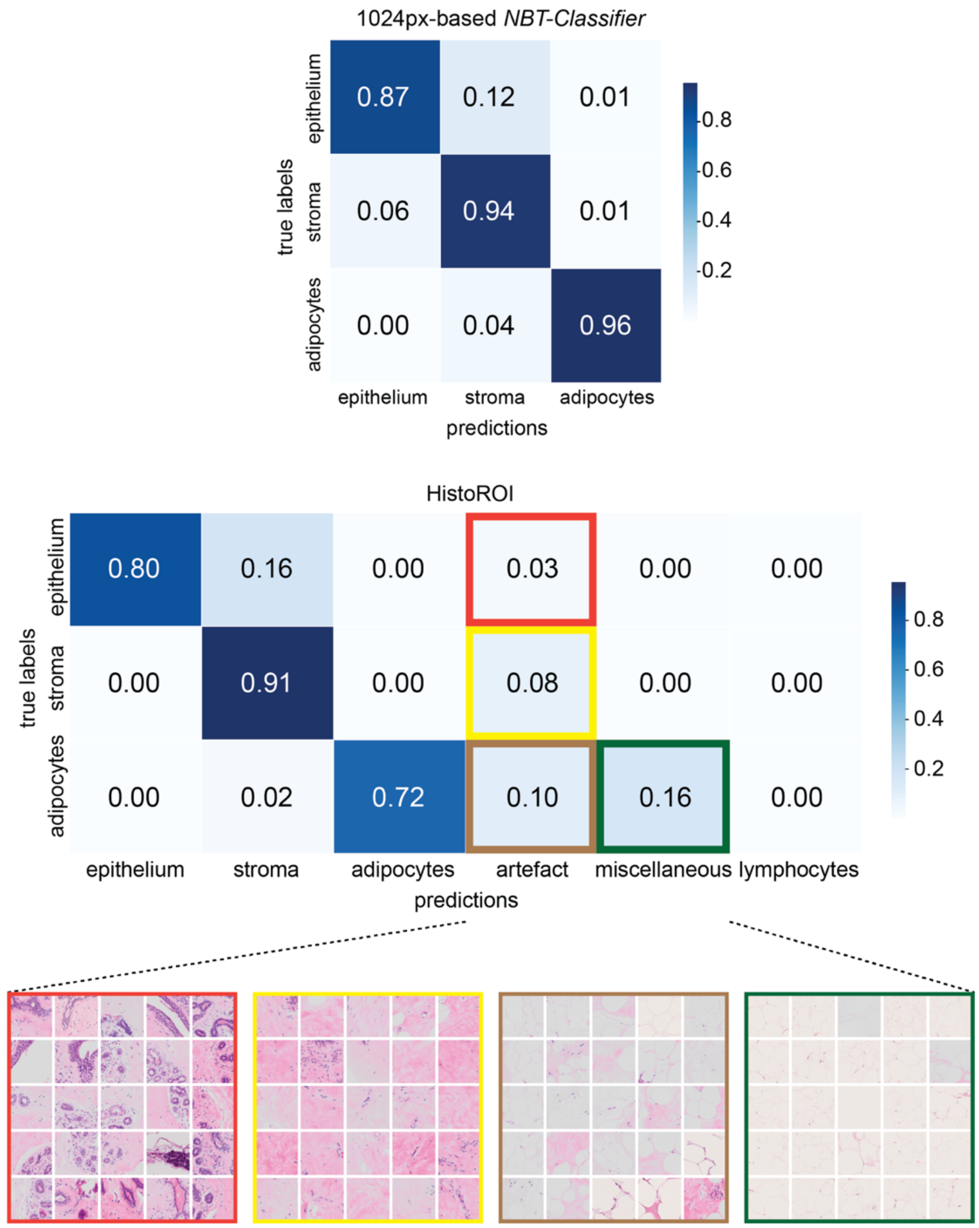

Supplementary Fig. 18 Proportional confusion matrices for the 1024px-based *NBT-Classifier* and HistoROI. For HistoROI, representative examples of epithelium and misclassified as artefact are indicated in red, stroma and misclassified as artefact are indicated in yellow, adipocytes and misclassified as artefact are indicated in brown, and adipocytes and misclassified as miscellaneous are indicated in green.

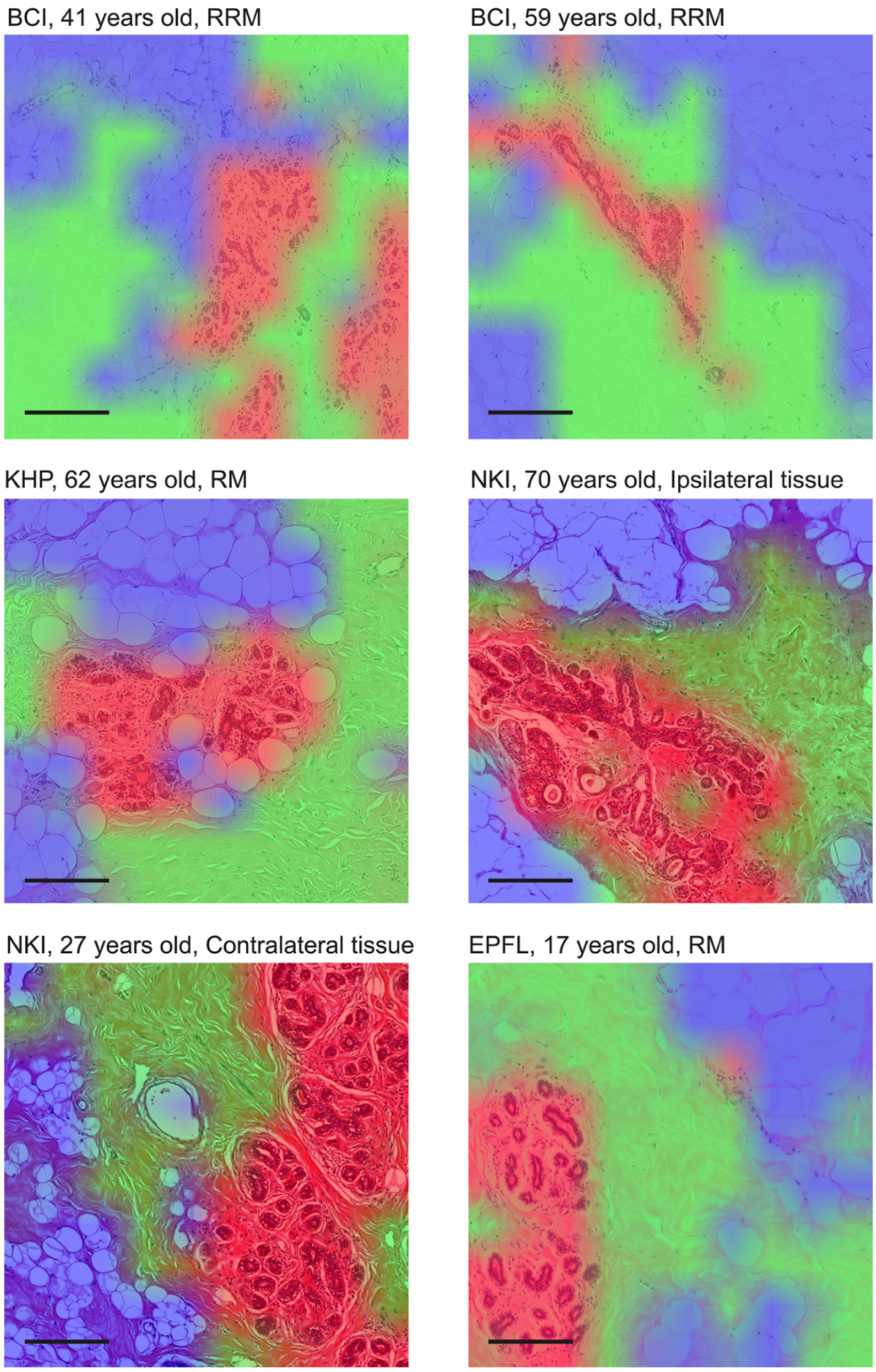

Supplementary Fig. 19 Representative regions with overlaid tissue probability heatmaps. Heatmaps were generated from NBTs with distinct patient ages and NBT sources, as indicated at the top of each figure. RRM: risk-reducing mastectomy; RM: reduction mammoplasty. Each heatmap clearly delineates the epithelium (red), stroma (green), and adipocytes (blue). Scale bar, 250 μm.

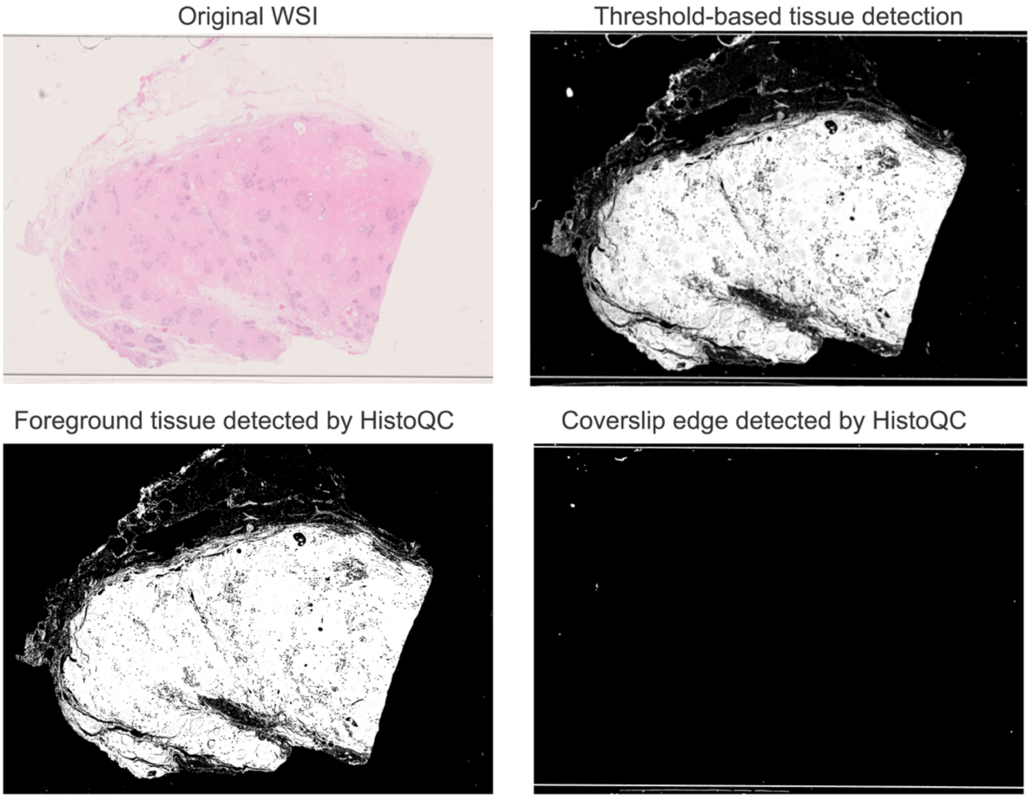

Supplementary Fig. 20 Illustration of foreground tissue detection using HistoQC. The top left image shows the original H&E-stained WSI of NBTs. The top right image shows the foreground tissue (in white) detected by the conventional Otsu threshold algorithm. Notably, the coverslip edge is incorrectly included as part of the foreground tissue. The bottom left image shows the foreground tissue (in white) detected by HistoQC, with the coverslip edge (in white) separately identified in the bottom right image.

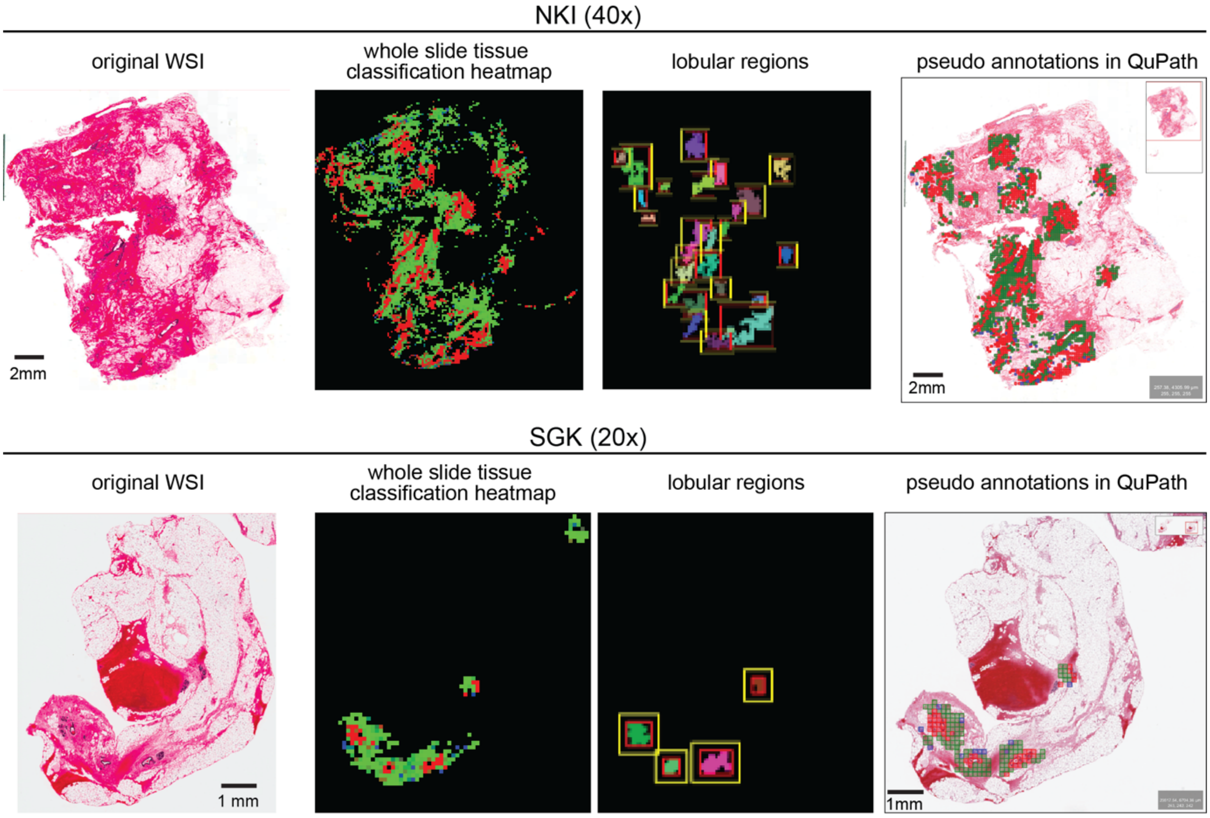

Supplementary Fig. 21 Examples illustrating the proposed *NBT-Classifier*-based WSI pre-processing pipeline. The top row shows a WSI example from the NKI cohort, scanned at 40x magnification, while the bottom row shows a WSI example from the SGK cohort, scanned at 20x magnification. From left to right, the images display the original WSI, the whole slide tissue classification heatmap, detected lobular regions (lobules are localised by the inner red boxes and peri-lobular regions are localised by the outer yellow boxes), and pseudo patch-level annotations (red for epithelium patches and green for stroma patches) visualised in QuPath v0.3.0.

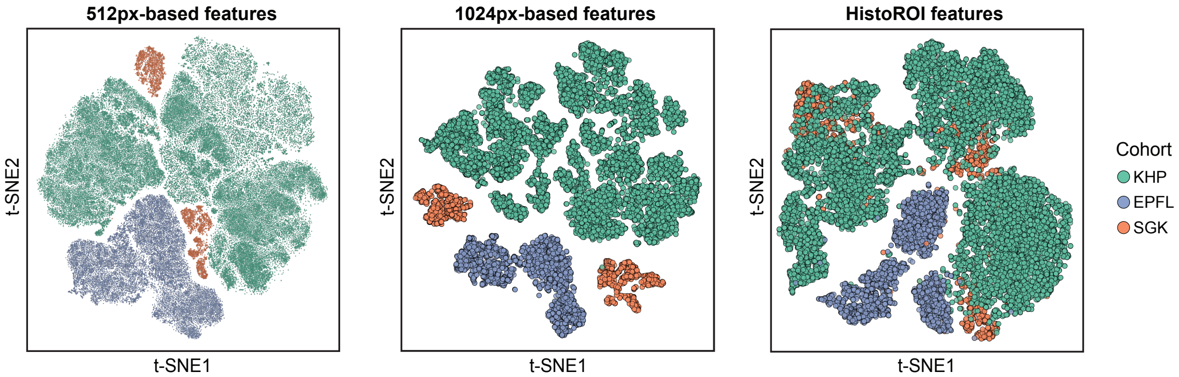

Supplementary Fig. 22 t-SNE visualisation of feature embeddings illustrating domain shift across cohorts. Feature embeddings are coloured by cohort for the 512px-based *NBT-Classifier* (left), 1024px-based *NBT-Classifier* (middle) and HistoROI (right).

Supplementary Tables

Supplementary Table 1. Overview of annotated WSI datasets of NBTs.

WSI id	Magnification	Data split	Cohort	Type of specimen	Patient age
LS13-03088N dc	40x	training	BCI	reduction mammoplasty	24
LS15-02541N-y					40
LS15-02672N-n					40
3046 B2					55
2282 B6				risk-reducing mastectomy	29
1097 A7					32
1438 K2					34
1130 D6					36
3154 LEFT PM					37
3239 Left PM					38
3031PM A6					39
1991PM A2					41
2243 A2					41
1524 A3					45
2204 B8					46
2975PM A3					59
T05-00863 A2 HE	40x	training	NKI	Contralateral NBT	27
T10-09302 A2 HE					32
T12-04313 A1 HE					36
T10-10050 A3 HE					40
T04-10775 A5 HE					47
T10-10006 A3 HE					56
T16-00449 A1 HE				Ipsilateral NBT	70
T13-05327 A4 HE				risk-reducing mastectomy	26
T18-08826 I1 HE					29
T19-02002 I1 HE					31
T15-08578 A1 HE					35
T15-00483 A3 HE					37
T13-04640 A2 HE					42
T13-04670 A1 HE					44
T13-04602 A4 HE					47
T14-01017 A3 HE					58
Human_195_s01_HE	40x	testing	EPFL	reduction mammoplasty	16
Human_103_s01_HE					17

Human_188_s01_HE					17
Human_129_s01_HE					18
Human_035_s01_HE					26
Human_016_s01_HE					28
Human_027_s01_HE					31
Human_172_s01_HE					32
Human_190_s01_HE					33
Human_131_s01_HE					39
19001626_FPE_3					22
18001177_FPE_1					23
17064108_FPE_1					29
18000951_FPE_2					30
17063396_FPE_5					32
17063838_FPE_2					37
17063839_FPE_3					37
17064113_FPE_4	40x	testing	KHP	reduction mammoplasty	38
19004666_FPE_9					41
17063968_FPE_3					45
17064240_FPE_4					47
17064241_FPE_7					47
17063451_FPE_3					59
17063504_FPE_6					62
17063106_FPE_3					69
17063107_FPE_4					69
K104210					19
K106560					29
K102595					30
K106776					32
K102518					40
K108158	20x	testing	SGK	core biopsies from healthy donors	46
K104934					50
K108301					51
K102425					52
K104724					60
K108355					72
K106822					74

Note: BCI: the Barts Cancer Institute in London (UK); NKI: the Netherlands Cancer Institute in Amsterdam (Netherlands); EPFL: the École Polytechnique Fédérale de Lausanne in Lausanne (Switzerland); KHP: the King's Health Partners Cancer Biobank in London (UK); SGK: the publicly available Susan G. Komen Tissue Bank; NBT: normal breast tissue.

Supplementary Table 2. Summary of annotated patch-level datasets.

Patch-level dataset	Data split	Cohort	Patch size	Annotation	Number of patches
NKI_512	training	NKI	512	epithelium	21,241
NKI_512	training	NKI	512	stroma	23,735
NKI_512	training	NKI	512	adipocytes	23,516
NKI_224	training	NKI	224	epithelium	110,639
NKI_224	training	NKI	224	stroma	123,497
NKI_224	training	NKI	224	adipocytes	122,668
NKI_1024	training	NKI	1024	epithelium	5,322
NKI_1024	training	NKI	1024	stroma	5,881
NKI_1024	training	NKI	1024	adipocytes	5,900
BCI_512	training	BCI	512	epithelium	9,144
BCI_512	training	BCI	512	stroma	14,214
BCI_512	training	BCI	512	adipocytes	15,273
BCI_224	training	BCI	224	epithelium	47,397
BCI_224	training	BCI	224	stroma	71,889
BCI_224	training	BCI	224	adipocytes	79,138
BCI_1024	training	BCI	1024	epithelium	2,281
BCI_1024	training	BCI	1024	stroma	3,212
BCI_1024	training	BCI	1024	adipocytes	3,768
KHP_512	external validation	KHP	512	epithelium	16,995
KHP_512	external validation	KHP	512	stroma	14,430
KHP_512	external validation	KHP	512	adipocytes	20,021
KHP_1024	external validation	KHP	1024	epithelium	4,039
KHP_1024	external validation	KHP	1024	stroma	3,327
KHP_1024	external validation	KHP	1024	adipocytes	4,467
EPFL_512	external validation	EPFL	512	epithelium	2,813
EPFL_512	external validation	EPFL	512	stroma	5,378
EPFL_512	external validation	EPFL	512	adipocytes	4,947
EPFL_1024	external validation	EPFL	1024	epithelium	654
EPFL_1024	external validation	EPFL	1024	stroma	1,149
EPFL_1024	external validation	EPFL	1024	adipocytes	1,068
SGK_256	external validation	SGK	256	epithelium	1,674
SGK_256	external validation	SGK	256	stroma	1,908
SGK_256	external validation	SGK	256	adipocytes	3,034
SGK_512	external validation	SGK	512	epithelium	416
SGK_512	external validation	SGK	512	stroma	472
SGK_512	external validation	SGK	512	adipocytes	741

Note: “train” refers to three-fold cross-validation, while “test” refers to external validation. The patch resolution is 0·5 μm per pixel for the SGK cohort and 0·25 μm per pixel for other cohorts. Patches were extracted without overlapping.

Supplementary Table 3. Summary of three-fold cross validation accuracies.

Cohort	Stain normalisation	CNN backbone	Patch size	Accuracy (mean)
NKI+BCI	none	MobileNet	512	95.75%
NKI+BCI	Macenko	MobileNet	512	97.32%
NKI+BCI	Vahadane	MobileNet	512	97.05%
NKI+BCI	Reinhard	MobileNet	512	97.72%
NKI+BCI	GAN	MobileNet	512	96.96%
NKI+BCI	none	ResNet50	512	96.86%
NKI+BCI	Macenko	ResNet50	512	96.84%
NKI+BCI	Vahadane	ResNet50	512	96.77%
NKI+BCI	Reinhard	ResNet50	512	97.25%
NKI+BCI	GAN	ResNet50	512	96.98%
NKI+BCI	none	InceptionV3	512	95.90%
NKI+BCI	Macenko	InceptionV3	512	96.25%
NKI+BCI	Vahadane	InceptionV3	512	96.23%
NKI+BCI	Reinhard	InceptionV3	512	96.32%
NKI+BCI	GAN	InceptionV3	512	96.86%
NKI+BCI	none	DenseNet	512	96.40%
NKI+BCI	Macenko	DenseNet	512	97.08%
NKI+BCI	Vahadane	DenseNet	512	96.49%
NKI+BCI	Reinhard	DenseNet	512	96.79%
NKI+BCI	GAN	DenseNet	512	96.58%
NKI+BCI	Reinhard	MobileNet	224	92.63%
NKI+BCI	Reinhard	MobileNet	512	95.53%
NKI+BCI	Reinhard	MobileNet	1024	97.03%

Supplementary Table 4. A summary of studies of NBTs with manual annotations in recent ten years.

Title	Source	Sample size	Manual annotations	Model
Deep learning assessment of breast terminal duct lobular unit involution: Towards automated prediction of breast cancer risk (10.1371/journal.pone.0231653)	benign breast biopsies	92 WSIs	Terminal duct lobular units (92 WSIs), acini and adipose tissue (50 WSIs)	Convolutional neural network
Automated quantification of levels of breast terminal duct lobular (TDLU) involution using deep learning (10.1038/s41523-021-00378-7)	benign breast biopsies	33 WSIs	Epithelium (and myoepithelium), extralobular stroma, intralobular stroma, adipose tissue, lumens of acini, and small calibre blood vessels (“capillaries”)	Convolutional neural network
Detection of lobular structures in normal breast tissue (10.1016/j.compbiomed.2016.05.004)	Reduction mammoplasty	9 WSIs	Lobules and ducts	Mixed methods
